# Epidemiological Characteristics of Infectious Diseases Among Travelers Between China and Foreign Countries Before and During the Early Stage of the COVID-19 Pandemic

**DOI:** 10.3389/fpubh.2021.739828

**Published:** 2021-11-03

**Authors:** Zheng Luo, Wei Wang, Yibo Ding, Jiaxin Xie, Jinhua Lu, Wen Xue, Yichen Chen, Ruiping Wang, Xiaopan Li, Lile Wu

**Affiliations:** ^1^Department of Neurology, Shanghai University of Medicine & Health Sciences Affiliated Zhoupu Hospital, Shanghai, China; ^2^Department of Infectious Disease Surveillance, Shanghai International Travel Healthcare Center, Shanghai, China; ^3^Department of Epidemiology, Second Military Medical University, Shanghai, China; ^4^Department of High Altitude Operational Medicine, Army Medical University, Chongqing, China; ^5^Office of Scientific Research and Information Management, Center for Disease Control and Prevention, Pudong New Area, Shanghai, China; ^6^Office of Scientific Research and Information Management, Fudan University Pudong Institute of Preventive Medicine, Pudong New Area, Shanghai, China; ^7^Office of Clinical Research Center, Shanghai Skin Disease Hospital, Shanghai, China; ^8^Department of Hepatobiliary Surgery, The Affiliated Hospital of Southwest Medical University, Luzhou, China

**Keywords:** infectious diseases, COVID-19, China, pandemic, Coronavirus

## Abstract

**Background:** International travel during the Coronavirus disease 2019 (COVID-19) pandemic carries a certain magnitude of infection risk both to travelers and their destination, which may be difficult to assess in the early stage. The characteristics of common infectious diseases of tourists may provide some clues to identify the high-risk travelers and protect susceptible population.

**Methods:** From among 48,444 travelers screened at Shanghai Port, we analyzed 577 travelers with 590 infectious diseases for age, sex, disease type, and World Health Organization (WHO) regions. We used the Joinpoint Regression Program to identify the average percent changes (APC) in the various trends among these individuals.

**Results:** Hepatitis B, syphilis, and HIV were the most common infectious diseases in travelers entering China, and Hepatitis B, pulmonary tuberculosis, and syphilis in Chinese nationals traveling abroad (overall detection rates, 1.43 and 0.74%, respectively; *P* < 0.05). Africa (2.96%), the Americas (1.68%), and the Western Pacific (1.62%) exhibited the highest detection rates. This trend did not decrease since the COVID-19 pandemic (*P* > 0.05) and rather showed an upward trend with increasing age [APC 95% CI = 5.46 (3.41,7.56)%, *P* < 0.05]. However, there were no evident trends in monthly infection rates of travelers exiting and entering China from different WHO regions (all *P* > 0.05).

**Conclusion:** Travelers always carry a transmission risk of common infectious diseases. It may be reasonable to adjust strategies for airport screening and quarantine according to the age and departure area of travelers to prevent and control new infectious diseases.

## Introduction

With economic globalization and continuous expansion of human activities, the space-time migration patterns among people and between people and animals have gradually changed. Due to the frequent interaction of humans with animals, the boundaries keeping various known or unknown viruses in wildlife from invading the human ecosystem have gradually disappeared (1, 2). Human infections from viruses that have undergone a long period of unknown natural selection and evolution in animals may lead to new infectious diseases and bring challenges of unforeseen magnitude to human health (3).

The detection of emerging infectious diseases is challenging, and this limitation makes their control difficult due to the occult transmission (4, 5). For example, in the early stages of COVID-19 pandemic, nearly 50% of COVID-19 cases were not detected in airport screening (4). In addition, the COVID-19 infection data issued by World Health Organization (WHO) is also limited by the release policies for epidemic data and detection strategies in different regions (6). Therefore, we need more indicators to assess the transmission risk of different travelers in order to identify the high-risk travelers, then we can increase the number of airport and post entry screening for those travelers from high-risk regions, and appropriately increase the days of quarantine, so as to reduce the transmission risk (7). Meanwhile, HIV, syphilis, tuberculosis, and other common infectious diseases are often monitored by the entry–exit inspection and quarantine departments of each country. This can be done effectively as the pathogens of these diseases are clear and established detection methods are available (7, 8). Although the source and route of transmission of different infectious diseases from infected people are different, the human-to-human spread of viruses usually occurs *via* droplets, sweat, blood, and other body fluids through contact or airborne transmission (9, 10). In particular, understanding the epidemic characteristics of infectious diseases that are common among international travelers will help understand the regional and population differences thereof, understand the demographic and geographical characteristics of infectious patients leaving and entering China, and define the infectious disease prevention and control level of different countries.

In this study, we analyzed the infectious disease disparities in WHO regions or age groups of entry-exit travelers at Shanghai Port before and after Jan 31, 2020 [considering Jan 31 as the time of COVID-19 being identified as a Public Health Emergency of International Concern (PHEIC) by the WHO] (11), and evaluated the temporal trends of the overall detection rate of common infectious diseases and the detection rate of each common infectious disease of Chinese nationals leaving China and travelers entering China by using the joinpoint regression model (12, 13), which may provide some clues for identifying the high-risk travelers (10). The results will help us determine whether the number of common infectious diseases is decreasing due to the decrease in the number of international travelers, or whether the infection rate of international travelers is reduced due to the spillover effect of prevention and control measures during the early stage of COVID-19 pandemic, ascertain the characteristics of infectious diseases in entry-exit travelers coming to China from different regions of the world before and during the early stage of COVID-19 pandemic as the evaluation indicators and provide a reference for discussing the modes of international transmission of emerging infectious diseases. This is particularly true for global cooperation in the prevention and control of COVID-19, which may be the next common infectious disease in human beings (14).

## Materials and Methods

### Data Source

Data in this study were acquired from Shanghai International Travel Healthcare Center in Shanghai Port, including the airport and seaport. This center is responsible for the legal work of health management and infectious disease monitoring of entry–exit personnel who plan to stay in the place of entry for more than 1 year.

### Study Design and Samples

To evaluate the characteristics of the infectious diseases of entry–exit travelers at Shanghai Port from Jan 1, 2019 to Apr 30, 2020, we conducted a retrospective cohort study by collecting data from the surveillance system in Shanghai International Travel Healthcare Center. During this period, data were collected from 48,444 individuals who were screened for common infectious diseases, including syphilis, HIV, pulmonary tuberculosis, malaria, hepatitis B, hepatitis C, hepatitis E, and infectious comorbidities.

We used the overall detection rate of common infectious diseases and the detection rates of each common infectious disease to compare the disparities between travelers exiting and entering China by age (including each 5-year age group encompassing 15–70 years of age as well as the age groups of ≤ 14 years and >70 years), sex (male and female), disease types, entry–exit types (exit/entry), and the WHO region of the nationality of the passenger (South-East Asia, Africa, the Americas, Europe, the Western Pacific, and Eastern Mediterranean and Other territories).

### Statistical Analysis

The detection rates of different groups were calculated and shown as percentages. They were compared according to the Poisson approximation method (15).

Joinpoint regressions have been widely used to analyze potential changes in trends (16). The time series were modeled using a few continuous linear segments, and the weighted sum of squared errors and the choice of the number of joinpoints were minimized on the basis of permutation tests (17). Compared to other regression methods used to investigate trends to find the best-fit line through years of data, the joinpoint analysis tests whether a multi-segmented line is a significantly better fit than a straight or less-segmented line (18, 19). Meanwhile, joinpoint analysis provides a much clearer picture of what is happening during a distinct period than a single summary trend statistic (20). Therefore, the study introduced the Joinpoint Regression Program 4.0.4 (source: https://surveillance.cancer.gov/joinpoint/) and expressed the changes as average monthly percentage change and average percentage change.

A Z test was used to assess whether the average monthly percentage change or average percentage change was statistically different from zero (19). All statistical analyses were performed using the Statistical Package for the Social Sciences software version 20.0 (SPSS, Inc., Chicago, IL.). *P*-value <0.05 was considered statistically significant.

## Results

### Baseline Characteristics

**Figure 1:**
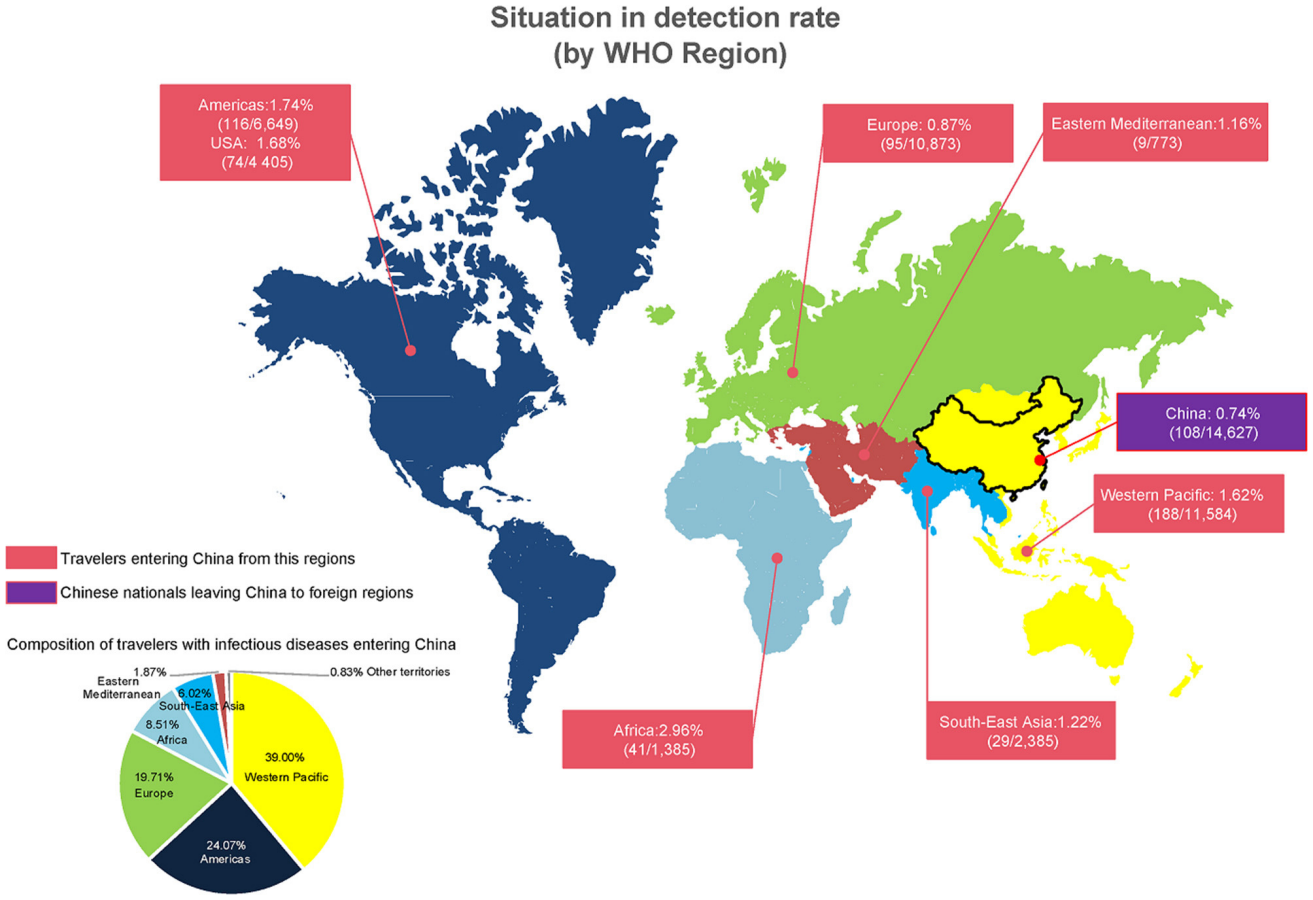
Regional differences in infectious disease cases among travelers at Shanghai Port, China, Jan 2019–Apr 2020.

During the study period, a total of 48,444 travelers were screened for infectious diseases, among whom 577 (1.19 %) travelers tested positive. Among these patients, 33,817 were travelers entering China and 14,627 were Chinese nationals leaving China, which accounted for 482 cases and 108 cases of infectious diseases, respectively (Table 1; Figure 1). The overall detection rate of common infectious disease in Chinese nationals leaving China was lower than that of travelers entering China (0.74 vs. 1.43%, *P* < 0.05).

**Table 1:** Characteristics of infectious diseases among travelers at Shanghai Port, China, Jan 2019–Apr 2020.

	**Cases (*n*, %)**	**Test population (*n*, %)**	**Detection rate (%)**	***P***
**Panel A: All travelers**				
**Status**				<0.001
Foreigner entry Shanghai	454 (76.94)	33,143 (68.42)	1.37	
Foreigner exit Shanghai	/	/	/	
Hong Kong, Macao and Taiwan Residence entry Shanghai	28 (4.75)	515 (1.06)	5.44	
Hong Kong, Macao and Taiwan Residence exit Shanghai	/	/	/	
Mainland Chinese entry Shanghai	0	159 (0.33)	0	
Mainland Chinese exit Shanghai	108 (18.31)	14,627 (30.19)	0.74	
**Sex**				<0.001
Male	386 (65.42)	27,095 (55.93)	1.42	
Female	204 (34.58)	21,349 (44.07)	0.96	
**Age**				<0.001
<40 years	286 (48.47)	37,026 (76.43)	0.77	
≥40 years	304 (51.53)	11,418 (23.57)	2.66	
**Period**				0.06
From Jan 1, 2019 to Jan 31, 2020	546 (92.54)	45,708 (94.35)	1.19	
From Feb 1, 2020 to Apr 30, 2020	44 (7.46)	2,736 (5.65)	1.61	
Total	590 (100.00)	48,444 (100.00)	1.22	
**Panel B: Travelers entering Shanghai, China**				
**WHO Regions**				<0.001
South-East Asia	29 (6.02)	2,385 (7.05)	1.22	
Africa	41 (8.51)	1,385 (4.10)	2.96	
Americas	116 (24.07)	6,649 (19.66)	1.74	
Europe	95 (19.71)	10,873 (32.15)	0.87	
Other territories	4 (0.83)	169 (0.50)	2.37	
Western Pacific	188 (39.00)	11,583 (34.25)	1.62	
Eastern Mediterranean	9 (1.87)	773 (2.29)	1.16	
**Period**				0.12
From Jan 1, 2019 to Jan 31, 2020	443 (91.91)	31,668 (93.65)	1.40	
From Feb 1, 2020 to Apr 30, 2020	39 (8.09)	2,149 (6.35)	1.81	
Total	482 (100.00)	33,817(100.00)	1.42	

*/, Data not shown*.

### Disparities in Age and WHO Region

The majority of travelers were 15–29 years old, with the peak age range being 20–24 years (9,640 persons). The majority of individuals with infectious diseases were aged 30–44 years, the average age was 40.60 ± 13.11 years old, and the peak age range was 40–44 years (81 cases). No child aged under 14 years was detected as having any infectious disease (Supplementary Table 1). The sex-specific detection rate of travelers segregated by months are shown in Supplementary Table 2. The detection rate of infectious diseases was higher in those aged ≥40 years than in younger individuals (*P* < 0.05; Table 1).

The proportion of male travelers was 55.93%, and the detection rate of infectious diseases was higher in men than in women (65.42 vs. 34.58%, *P* < 0.05; Table 1).

In the tested population, 33,143 travelers entering China accounted for 68.42% of cases, and the number of travelers entering and leaving China were higher from Jul to Sept 2019 and decreased after Jan 2020 to about 50% of what they were in the same period in 2019. The top 3 months with the highest recorded infectious diseases were Sept, Jul, and Apr 2019, and then, the number of infectious diseases detected decreased in 2020 to about 50% of that in the same period in 2019 (Supplementary Table 2).

In addition, there were no significant differences in the detection rates of travelers entering China before and during COVID-19 pandemic. Similarly, there were no significant differences in the detection rates of travelers entering China during Feb–Apr 2019 and during Feb–Apr 2020 vs. those leaving China during Feb–Apr 2019 and during Feb–Apr 2020 (all *P* > 0.05; Table 1, Panel A and Panel B; Supplementary Tables 3, 5). The number of travelers entering China and the monthly detection rates between Jan 31, 2020 and Apr 30, 2020 are shown in Supplementary Table 3. The numbers of travelers from each country or region are shown in Supplementary Table 4, and the detection rates of different infectious diseases are shown in Supplementary Table 5.

The top three original regions of travelers entering into China were the Western Pacific, Europe, and the Americas; however, the highest rate of infection was observed in travelers from Africa, followed by the Western Pacific, and the Americas, with all three regions collectively accounting for 71.58% of all cases (Figure 1). There were significant differences in the positive detection rate among people from different regions (*P* < 0.001; Table 1).

**Figure 2:**
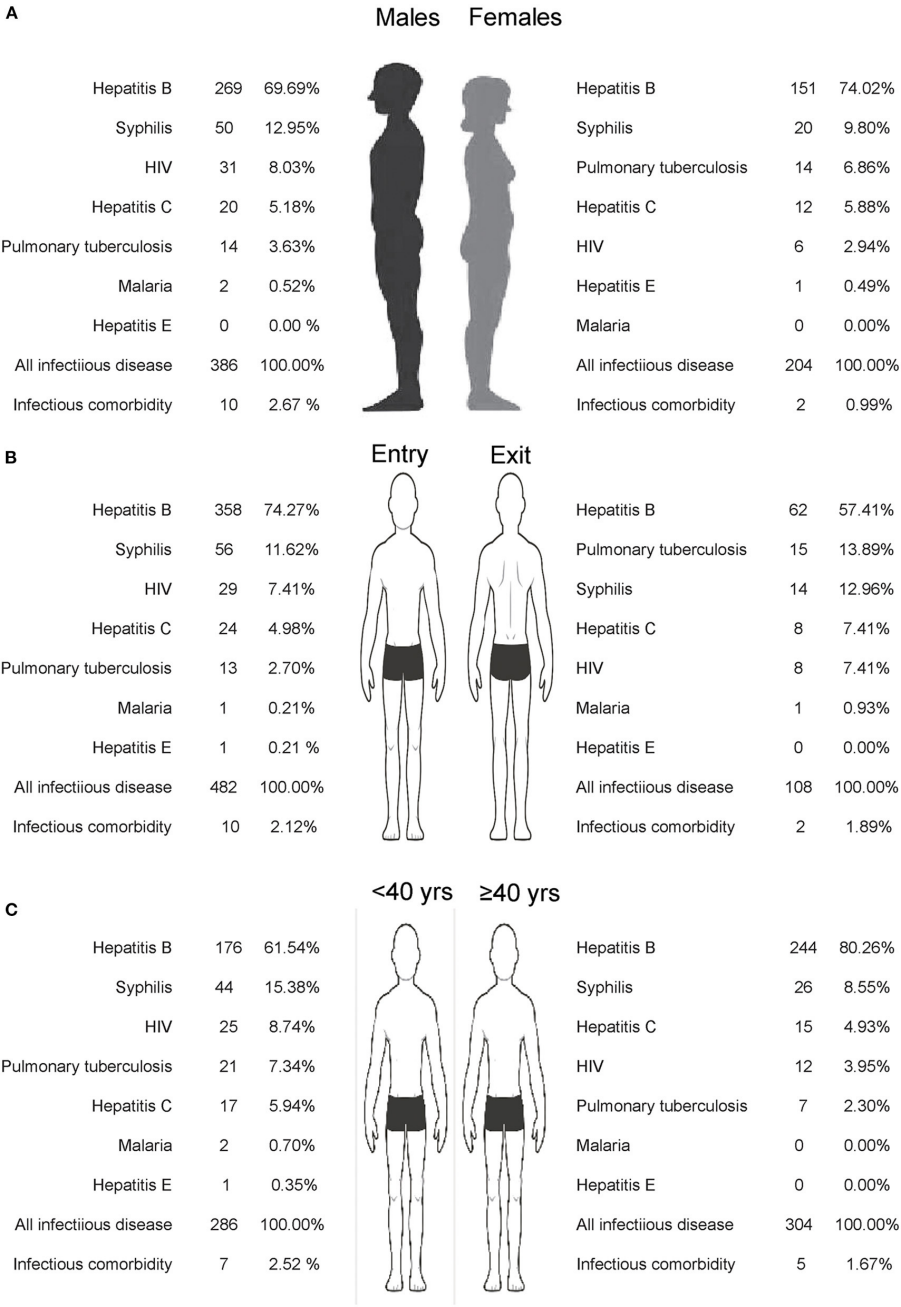
Detection spectrum of infectious diseases of travelers at Shanghai Port, China, Jan 2019–Apr 2020 **(A)** Sex; **(B)** Entry and exit population; **(C)** Age group.

The top three common infectious diseases were hepatitis B, syphilis, and HIV among men, travelers entering China, and travelers <40 years old, accounting for 90.67, 91.91, and 85.66% of all cases, respectively. Similarly, the top three common infectious diseases were (1) hepatitis B, syphilis, and pulmonary tuberculosis among women, accounting for 86.76% of all cases; (2) hepatitis B, pulmonary tuberculosis, and syphilis among Chinese nationals leaving China, accounting for 84.26% of all cases; and (3) hepatitis B, syphilis, and hepatitis C among travelers aged ≥40 years. There were statistically significant differences in the spectrum of infectious diseases detected in entry–exit personnel (entering China vs. leaving China, *P* = 0.001) in terms of sex (male vs. female, *P* = 0.03) and age (<40 years vs. ≥40 years, *P* < 0.001; Figures 2A–C).

### Trend Analysis

**Figure 3:**
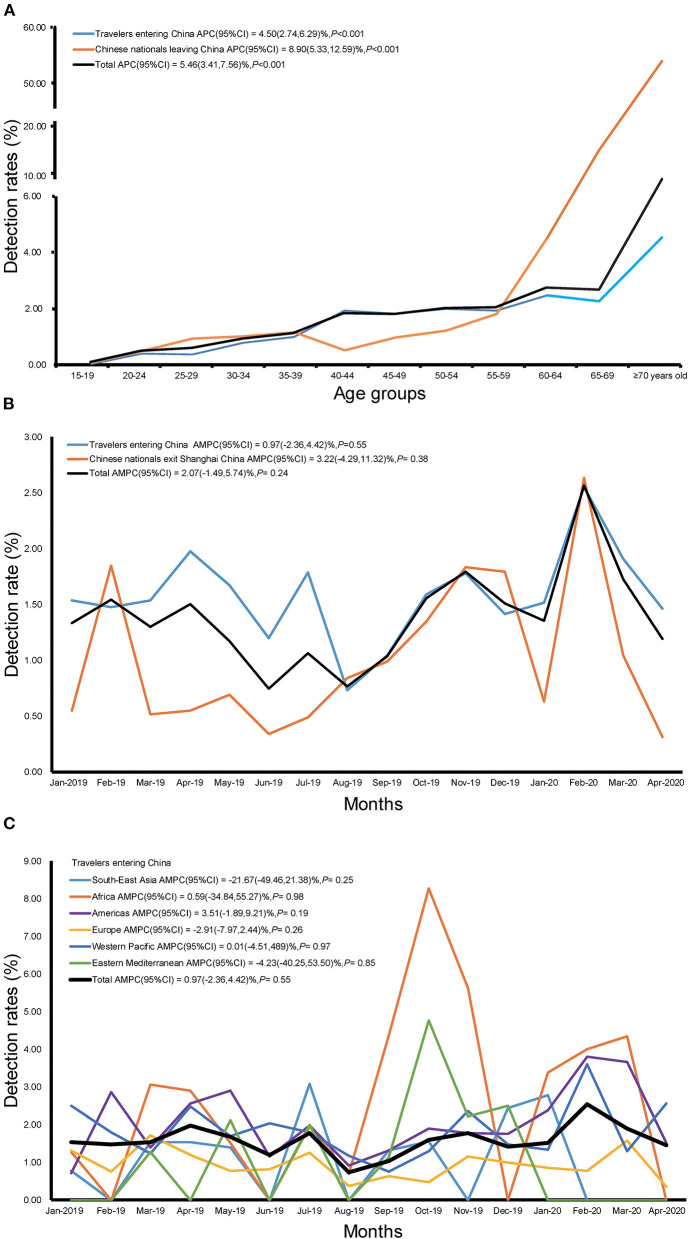
Trend of detection rate of infectious diseases among travelers at Shanghai Port, China, Jan 2019–Apr 2020 **(A)** Age trends; **(B)** Time trends; **(C)** Time trends in each region.

For all travelers, the average detection rate of an infectious disease was 1.22%; the lowest detection rate was 0.12% in the 15–19 years age group; and the highest detection rate was 9.18% in individuals over 70 years of age. With increasing age, there were a significant overall detection rate [APC 95% CI = 5.46 (3.41,7.56)%, *P* < 0.05] of common infectious diseases in travelers exiting and entering China throughout the entire period. In addition, the overall detection rate in travelers entering China showed a significant increase of 4.50% (95% CI: 2.74, 6.29%, *P* < 0.001), and the overall detection rate in Chinese nationals leaving China showed a significant increase of 8.90% (95% CI: 5.33, 12.59%, *P* < 0.001). Figure 3A shows the trend of detection rates associated with different age groups for all travelers, travelers entering China, and Chinese nationals leaving China. Supplementary Table 1 shows these detection rates separately for men and women.

The detection rate of infectious diseases with increasing time indicated a fluctuating trend without an obvious increasing or decreasing trend for travelers entering China and for Chinese nationals leaving China on a monthly basis (all *P* > 0.05; Figure 3B; Supplementary Table 2). In addition, there was no statistically significant difference in the monthly data of detection rates of infected individuals leaving China (to anywhere), entering China (from anywhere), or entering China (from each WHO region; all *P* > 0.05; Figure 3C; Supplementary Table 3).

## Discussion

As the number of emerging infectious diseases is increasing, the current global situation of infectious diseases is becoming increasingly severe (21, 22). As exemplified by the COVID-19 pandemic, the spread of epidemic diseases not only seriously endangers human health and national economic development but also affects social stability (23). To actively respond to an infectious disease epidemic, the top priority should be aimed at preventing the worldwide spread of the epidemic and at strengthening the prevention and control lines of infectious diseases at ports to prevent the import of overseas cases (24). Among such measures, strategies should be developed to follow closely the global epidemic situation, strengthen epidemic control and risk analysis, improve port health inspection and quarantine protocols, and ensure the surveillance of entry and exit of the infectious diseases (25, 26).

To achieve the above-mentioned goals, it is of primary importance that travelers, who are very important to economic development, understand the spread of infectious diseases and the epidemic characteristics of infectious diseases so as to follow the emergency epidemic prevention policies formulated by various countries or regions (27, 28). Our results showed that the overall detection rate of common infectious diseases among travelers entering and leaving Shanghai Port of China did not change significantly with time and climate change. Furthermore, the detection rate of different diseases did not significantly differ before and during the early stage of the COVID-19 pandemic. Globally, even though some infectious diseases are seasonal, the overall detection rate of imported and exported common infectious diseases in Shanghai, China has not changed significantly in recent times. Therefore, the Customs officials need to pay attention to the prevention of infectious diseases in both peak season and off-season travel because the enhancement of prevention and control measures to prevent novel emerging infectious diseases has not been able to reduce the infection proportion of common infectious diseases among travelers. Meanwhile, in early stages of the spread of new infectious diseases (e.g., COVID-19), the infection risk may not be completely eliminated by the reduction in the number of travelers alone because of insufficient means to detect the novel infection (3). Furthermore, we would like to particularly emphasize the finding that the proportion of infectious diseases carried by Chinese residents leaving the country was lower than that of infectious diseases carried by people of other regions in the world entering China. This shows that China's public health policy, which implements the principle of modern prevention first, is an effective approach (26). Furthermore, these results show that China's infectious disease prevention and control system operates well and with the aim to prevent and control the infection. This approach thus guarantees the control of the COVID-19 pandemic within the country and is also a basic demonstration of how to prevent the spread of new infectious diseases to other countries (29). Identifying the basic features of common infectious diseases can help people reach a consensus and avoid rumors that result in prejudices (10, 30).

We also need to recognize the regional diversities of infectious diseases. Our results show that travelers coming from Western Pacific region have the largest number of detected infectious cases, which can be attributed to this region being responsible for the largest number of travelers coming to China. Furthermore, the travelers entering Shanghai Port of China from Africa have the highest detection rate of infectious diseases, which may be attributed to the lack of local health resources and the relatively higher infection rate in Africa (31). These findings suggest that in the context of international exchanges, the prevention and control of infectious diseases endemic to Africa is an indispensable step in the prevention of global spread of these diseases. These results also suggest that the COVID-19 control in Africa is related to the public health and safety of the world (32–34).

It is also indispensable to understand the international infectious disease spectrum in different populations. The results of this study revealed that in men, the detection rate of HIV was in the third place and tuberculosis was in the fifth place, whereas in women, tuberculosis was in the third place and HIV was in the fifth place. Nevertheless, the absolute number and detection rate of HIV infection were higher in men than in women, which may be associated with the higher exposure level of gay men to HIV (35–37). The results of this study showed that the infection rate of infectious diseases in travelers aged >15 years increased with age, and the highest detection rate was noted in travelers aged >70 years. The detection rate of common infectious diseases among Chinese residents aged >70 years was 57.14% (4/7). Conversely, these findings may be attributed to the bias caused by the fact that the majority of international tourists were younger than 40 years of age (76.43%). The population aged >60 years only accounted for 2.56% of the total population, and the population aged >70 years was not sufficient (98 people) for data analysis. This also suggests that the representativeness of the population aged >70 years was not large enough. By contrast, the findings in this age group may also be related to the opportune accumulation of infectious diseases as this population lived through a time when healthcare policies were weak and vaccines were not popular. These policies may have increased the exposure level of these individuals to pathogens and reduced the body's immunity (38–40). However, the high detection rate of common infectious diseases in the higher age group cannot be underestimated. More than 80% of the cases in patients aged >40 years exhibited hepatitis B, which indicated that the prevention and treatment of hepatitis B among travelers in this age group is very important. This also highlights the protective effect of hepatitis B vaccine on human beings after its introduction, as young travelers were completely free from hepatitis B infection (41). In terms of the entry–exit population, syphilis and HIV among foreign travelers ranked as the second and third most common infections, respectively, while tuberculosis and syphilis among Chinese nationals ranked as the second and third most common infections, respectively. Therefore, it maybe helpful for destination countries to carry out targeted preventive and control measures for travelers exiting and entering China according to the infection spectrum of different common infectious diseases (42–45).

Finally, it is requisite to understand the transmission characteristics of common infectious diseases to overcome the fear of new unknown infectious diseases (6, 46). Many new, unknown infectious diseases will eventually transform into common infectious diseases and will require researchers to identify their characteristics and pathogenic mechanism to make medicines available (47). Thus, our results will help countries adjust strategies for airport screening and quarantine according to the age and departure area of travelers to prevent and control new infectious diseases (48).

Our study has some limitations. First, due to different basic reproduction number (R0) and source and route of transmission, it is difficult to design control and prevention strategies against emerging infectious diseases (e.g., COVID-19) on the basis of the detection rates of common infectious diseases in international travelers (26, 49). However, as our knowledge about COVID-19 has increased with time, it can be used as a reference for the prevention and control of other infectious diseases in the future. Second, there were not enough months of monitoring data since the COVID-19 pandemic started because China has taken strict prevention and control measures to greatly reduce the traffic of international travelers after May 2020 (9). In addition, there were no data of the COVID-19 infection in people having common infectious diseases because we had no authority to detect the infection of common infectious diseases of travelers transferred to the hospital immediately due to the diagnosis of COVID-19. More data will be available only when international travel normalizes in the future; however, the future is currently unpredictable. Finally, the infectious disease monitoring of the Customs can only target the travelers who are expected to enter the country for more than 1 year; therefore, a large number of individuals traveling for a shorter period are not included in the monitoring system. This may lead to underestimation of the infection rates because individuals who travel a lot are more likely to harbor an infectious disease (5, 50). However, it is gratifying that all travelers are currently being tested for COVID-19 regardless of their stay duration.

Generally, the policy of prevention and control of the international spread of infectious diseases may be based on already known common infectious diseases. Additionally, novel emerging infectious diseases often spread in hiding in the early stages until detection methods for the causative agent are established. We should therefore focus on the regional and population diversities rather than relying on time or climate changes to eradicate infectious diseases. Overall, it is important to strengthen mutual cooperation in the prevention and control of infectious diseases among countries and to restore world order and safe public health.

## Data Availability Statement

The original contributions presented in the study are included in the article/Supplementary Material, further inquiries can be directed to the corresponding author/s.

## Ethics Statement

Our study did not involve any intervention in human participants. The surveillance protocol was approved by the Ethical Committee of Shanghai International Travel Healthcare Center. Verbal informed consent was obtained from each subject. Individual information was de-identified prior to analysis. Strict confidentiality of individual data was practiced during the entire study.

## Author Contributions

XPL, LLW, and RPW designed the research. ZL, WW, YCC, JHL, and WX collected the data. ZL, WW, and YBD performed the research and analyzed the data. XPL, LLW, and ZL wrote the paper. XPL, LLW, RPW, and JXX provided critical revisions. All authors contributed to the article and approved the submitted version.

## Funding

This work was supported by a grant from the Key Disciplines Construction Foundation of Health Commission of Shanghai Pudong New District of China (PWZxk2017-25 to ZL), a grant from the project of Shanghai University of Medicine & Health Sciences Affiliated Zhoupu Hospital (zpxm-2019b-05 to ZL), a grant from the Reserve Discipline Leader Talents Training Program of the Pudong Center for Disease Control and Prevention (PDCDC-HBXD2020-05 to XPL), a grant from Shanghai Public Health System Construction Three-year Action Plan Outstanding Youth Talent Training Program (GWV-10.2-YQ43 to YCC), a grant from the Research Program of Shanghai Sports Bureau (20Q001 to RPW), Key disciplines of public health in Shanghai (GWV-10.1-XK17 to YBD), COVID-19 project of National Natural Science Foundation of China (82041022 to YBD), a grant from the National Natural Science Foundation of China (Grant No. 81803294 to JXX).

## Conflict of Interest

The authors declare that the research was conducted in the absence of any commercial or financial relationships that could be construed as a potential conflict of interest.

## Publisher's Note

All claims expressed in this article are solely those of the authors and do not necessarily represent those of their affiliated organizations, or those of the publisher, the editors and the reviewers. Any product that may be evaluated in this article, or claim that may be made by its manufacturer, is not guaranteed or endorsed by the publisher.

## Supplementary Material

Supplementary Table 1 Detection of age-specific infectious diseases among travelers at Shanghai Port, China, Jan 2019–Apr 2020.

Supplementary Table 2 Detection of infectious diseases among male and female travelers at Shanghai Port, China, Jan 2019–Apr 2020.

Supplementary Table 3 Detection of infectious diseases in various regions of travelers at Shanghai Port, China, Jan 2019–Apr 2020.

Supplementary Table 4 Distribution and detection of infectious diseases among travelers at Shanghai Port, China, Jan 2019–Apr 2020.

Supplementary Table 5 Detection of infectious diseases in travelers entering or leaving Shanghai Port, China, Jan 2019–Apr 2020.
