# Supplementary material for: Epidemiological Characteristics of Infectious Diseases Among Travelers Between China and Foreign Countries Before and During the Early Stage of the COVID-19 Pandemic
Source: Front Public Health. 2021 Nov 3;9:739828. doi: 10.3389/fpubh.2021.739828 (PMC8634889; doi:10.3389/fpubh.2021.739828)
Supplement: Supplementary file 1 [file Data_Sheet_2.ZIP › Appendix 1_Consent Form/Consent Form.docx]

Applicant’s Full Name Passport or ID No.

According to <The Frontier Health And Quarantine Law of P.R.C.>, <Specific Rules For Enforcing The Frontier Health And Quarantine Law Of The People’s Republic Of China On Control Of The Entry And Exit Of Aliens> and related documents issued by the Ministry Of Health and the Ministry of Public Security Of China, any foreigners (Including Hong Kong and Macao residents, Chinese citizens from Taiwan Province, Chinese mainland citizens setting overseas) applying to settle down, work or study in China for one or more than one year are required to receive health examination, any Chinese citizens approved to work in, study in, visit or immigrate into another country for more than one year are required to receive health examination.

You are supposed to receive physical examinations, including clinical examination (exams in medicine and surgery, ENT, C-ray, EKG and ultrasound) and blood tests (HBsAg, anti-HCV, anti-HIV, and treponemal specific antibody, any others tests according to outbreak of infectious diseases requested by General Administration of Quality supervision, Inspection and Quarantine and the Health Ministry of China etc.) or urine tests.

Citing privacy and confidential information, please unseal the report in **person and keep it properly** when you receive the health report.

I have read and understood the above statement. I hereby consent to undergo the health examination and will pay the cost. Meanwhile, I apply for the certificate of health examination.

**Signature:**   **Date:**
