# Supplementary figures and images for: Epidemiological Characteristics of Infectious Diseases Among Travelers Between China and Foreign Countries Before and During the Early Stage of the COVID-19 Pandemic
Source: Front Public Health. 2021 Nov 3;9:739828. doi: 10.3389/fpubh.2021.739828 (PMC8634889; doi:10.3389/fpubh.2021.739828)

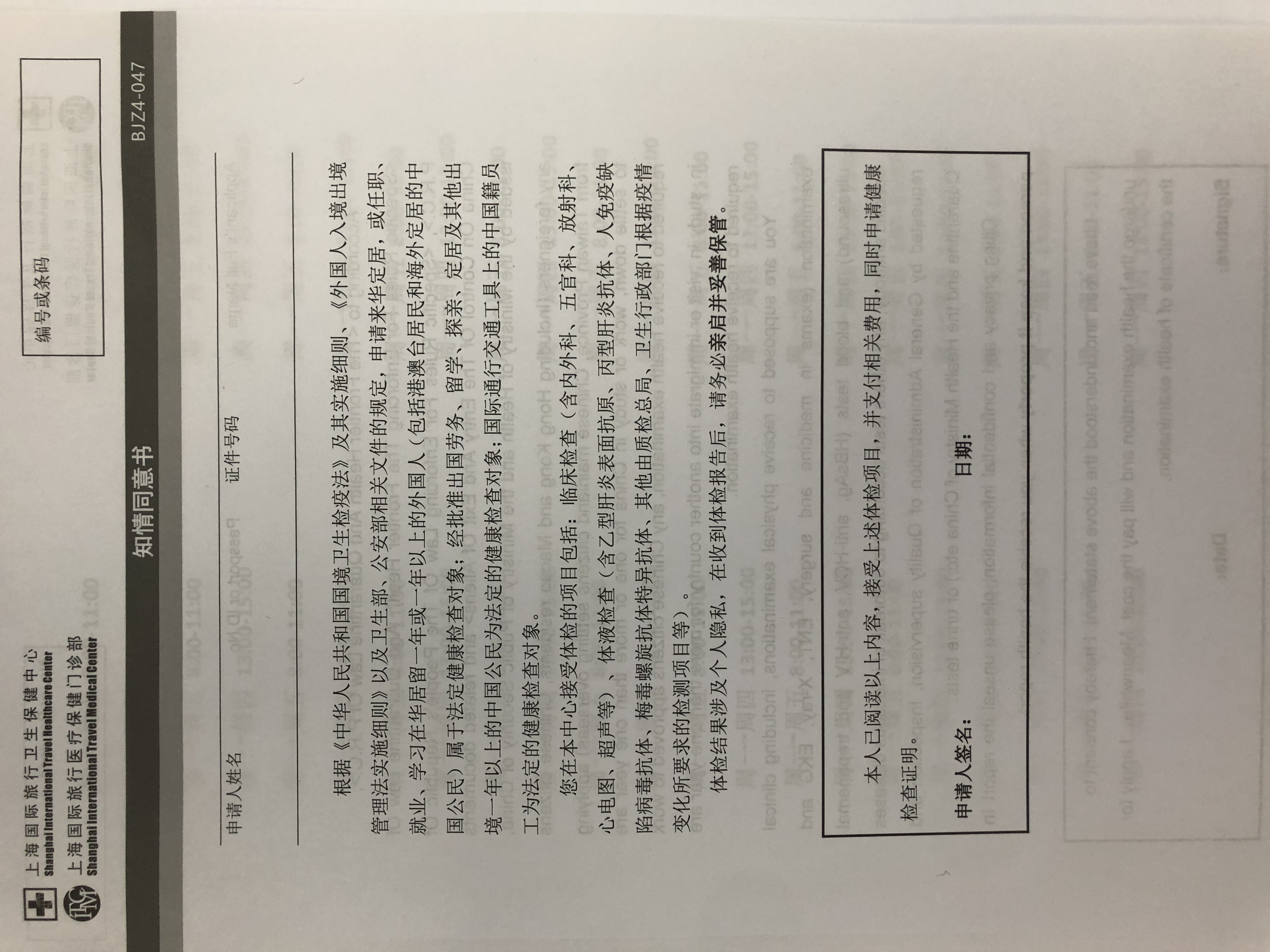

Supplement: Supplementary file 1 [file Data_Sheet_2.ZIP › Appendix 1_Consent Form/Consent Form (in Chinese).jpg]

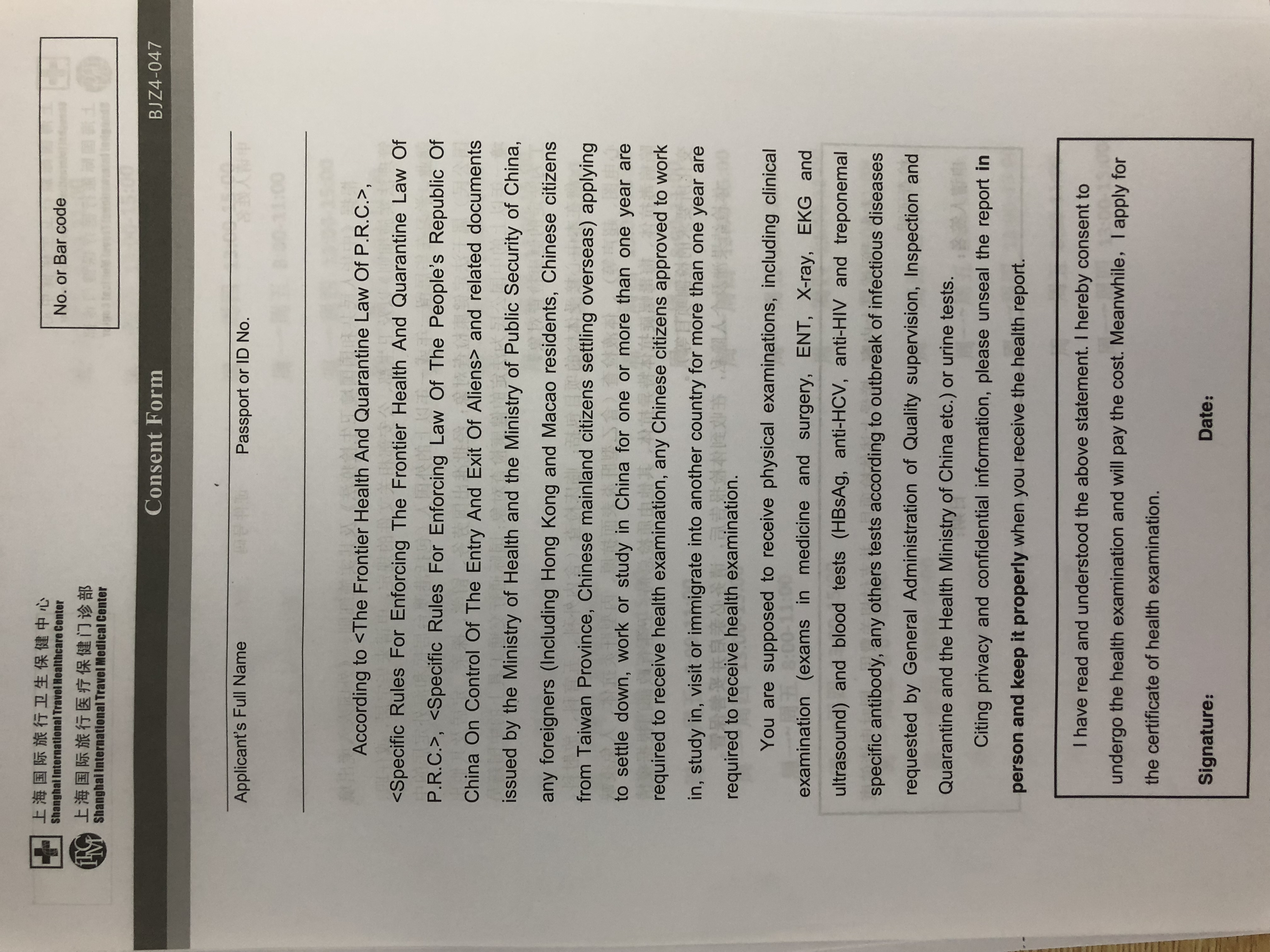

Supplement: Supplementary file 1 [file Data_Sheet_2.ZIP › Appendix 1_Consent Form/Consent Form(in English).jpg]
